# Describing methods and interventions: a protocol for the systematic analysis of the perioperative quality improvement literature

**DOI:** 10.1186/2046-4053-3-98

**Published:** 2014-09-05

**Authors:** Emma Jones, Nicholas Lees, Graham Martin, Mary Dixon-Woods

**Affiliations:** 1 Department of Health Sciences, University of Leicester, 22-28 Princess Road West, Leicester LE1 6TP, UK; 2 Department of Anaesthesia, Royal Brompton and Harefield NHS Foundation Trust, Hill End Road, Harefield, Middlesex UB9 6JH, UK

**Keywords:** Quality improvement, Perioperative care, Surgery, Interventions, Reporting, Quality of care, Description

## Abstract

**Background:**

Quality improvement (QI) methods are widely used in surgery in an effort to improve care, often using techniques such as Plan-Do-Study-Act cycles to implement specific interventions. Explicit definition of both the QI method and quality intervention is necessary to enable the accurate replication of effective interventions in practice, facilitate cumulative learning, reduce research waste and optimise benefits to patients. This systematic review aims to assess quality of reporting of QI methods and quality interventions in perioperative care.

**Methods:**

Studies reporting on quality interventions implemented in perioperative care settings will be identified. Searches will be conducted in the Ovid SP version of Medline, Scopus, the Cochrane Central Register of Controlled Trials, the Cochrane Effective Practice and Organisation of Care database and the related articles function of PubMed. The journal *BMJ Quality* will be searched separately. Search strategy terms will relate to (i) surgery, (ii) QI and (iii) evaluation methods. Explicit exclusion and inclusion criteria will be applied. Data from studies will be extracted using a data extraction form. The Template for Intervention Description and Replication (TIDieR) checklist will be used to evaluate quality of reporting, together with additional items aimed at assessing QI methods specifically.

**Systematic review registration:**

PROSPERO http://CRD42014012845

## Background

Quality Improvement (QI) methods are specially designed efforts and processes aimed at generating improvements in patient care [1]. Such methods include those based on Lean, Six Sigma, Plan-Do-Study-Act (PDSA) cycles, Total Quality Management and Continuous Quality Management, audit and feedback, and many others [2]. Guidance on reporting of QI studies [3] and of intervention delivery in evaluative studies [4-7] has been published. Surgery is an especially important area for quality improvement: an estimated 234 million surgical interventions are performed every year worldwide [8], yet it remains hazardous and prone to error and complication. An international drive to improve quality of care in surgery is now supported by initiatives such as the Centre for Global Surgery [9] and the Lancet Commission for Global Surgery [10]. Yet the quality of reporting of interventions in QI studies in surgery is unknown. This is an important problem, as it is increasingly recognised that explicit descriptions of interventions are necessary to ensure that successful interventions can be replicated in practice, to avoid research waste, to facilitate cumulate learning and to ensure that patients gain the best possible benefits from any learning from QI studies [11,12]. We seek to adopt and adapt the Template for Intervention Description and Replication (TIDieR) checklist [4] to evaluate the quality and completeness of reporting of studies of quality improvement interventions in perioperative care.

Perioperative care is a process encompassing care received before, during and after a surgical procedure [13]. The translation of successful QI strategies into surgical practice has the potential to contribute towards ensuring the delivery of safe, high-quality, accessible and affordable surgery [2,14-18]. Systematic review has evaluated data generated by QI methods across cardiothoracic, colorectal [14], vascular, hepatobiliary and upper gastrointestinal specialties [2,15,16] and has reported measureable improvements across the whole perioperative journey including the preoperative period (reduction in time to surgery [16]), intraoperative period (reduction of sepsis [16]) and postoperative period (reduction of surgical site infection [14,16], central venous catheter infection [16] and venous thromboembolism [16]) Yet the QI literature in surgery has also been found to suffer from a range of problems including lack of explicit rationale, poor detail and overlapping components in the published descriptions of QI methods [2,19] and quality interventions [20]. The extent and quality of patient and public involvement (PPI) in surgical research are also unclear, despite recommendations that the Guidance for Reporting Involvement of Patients and Public (GRIPP) checklist be used in order to provide a quality assurance on the level of PPI reporting [21].

One problem in assessing the literature on quality improvement is a degree of conceptual and terminological confusion over the term ‘intervention’. The methods of improvement are sometimes referred to as interventions, yet so too are the quality interventions that such methods seek to implement. Thus, for example, the literature may use the term ‘intervention’ interchangeably to describe both application of the PDSA method and a quality intervention such as a checklist or ‘bundle’.

For purposes of this review, QI *methods* will be defined as the processes (such as PDSA cycles) which are typically intended to support the implementation of a quality intervention. Quality *interventions* will be defined as the individual components of care delivery which are implemented in order to achieve an improvement in the delivery of patient care. Quality interventions need to be described explicitly and precisely if it is to be possible to implement them. The parameters that might be used in describing such interventions include

• What (which materials, and activities should be used)

• Who (qualification type and competency)

• How (face-to-face, in a group, via the internet)

• Where (setting, infrastructure)

• When and how much (dose, timing, frequency, duration)

• Tailoring (personalisation)

• Modifications (changes during the course of the study)

• How well (what challenges were identified, e.g. dropouts or missing data)

### Aims

This systematic review aims to assess the completeness of reporting within the perioperative literature on QI methods and quality interventions and to identify which elements are most frequently missing.

## Methods

### Design

We will undertake a review of the published qualitative and quantitative surgical literature on QI. We will define QI methods as the processes which are usually intended to support implementation of the quality intervention such as PDSA cycles. We will define quality interventions as the individual components of care delivery which are selected in order to make an improvement (such as issuing checklists or care bundles).

### Eligibility criteria

This review will include

• All studies published between 1 January 2000 and 28 May 2014 to capture all papers indexed since the publication of the Institute of Medicine's ‘To Err is Human: Building a Safer Health System’ report [22], which highlighted the importance of systems-based interventions to address quality and safety problems

• All surgical specialities

• Adult surgical services

• Elective and emergency (trauma) surgery

• Primary and secondary care, because hospital stay is just one aspect of the surgical patient's whole pathway [23]

• Studies using both validated and unvalidated measures

• Studies meeting the criteria within the QI taxonomy generated by Shojania et al. [24] (Table 1)

**Table 1:** Quality improvement taxonomy

**QI strategy**	**Definition**	**Methods**	**Surgical examples**
1. Provider education	Dissemination of information	Educational outreach visits	Component separation training and recurrence rates
		Distribution of educational material	Cadaveric training and surgeon confidence
2. Provider reminder systems	Any ‘clinical encounter-specific’ information intended to prompt a clinician to recall information or consider a specific process of care	Decision aids	MEWS
		Reminders	The WHO surgical safety checklist
3. Patient reminders	Any methods of encouraging patient compliance to self-management	Appointment reminders	SMS exercise reminders before bariatric surgery
4. Promotion of self-management	Access to a resource that enhances the patients' ability to manage their condition	BP devices	Follow up phone calls with recommended adjustments to care
		Fit Bits/pedometers	
5. Audit and feedback	Any feedback of clinical performance	PROMs	Percentage of patients achieving target LOS
		LOS	
		Morbidity and mortality	
6. Patient education	Dissemination of information	Distribution of educational material	Tri-modal pre-habilitation programme compliance and effect on LOS
		Individual or group sessions	
7. Organizational change	Any change in organizational structure	Multidisciplinary teams	Changes to staff rota to facilitate early patient mobilization after elective arthroplasty
		Communication	
		Health records	
8. Financial, regulatory, or legislative incentives	Any financial bonus, reimbursement or provider licensure scheme	Positive or negative incentives for providers or patients	18-week wait target for elective orthopaedic surgery
9. Facilitated relay of clinical data to providers	Transfer of clinical information from patients to the provider when data was not collected during a patient visit	Telephone call	Relay of BP measurements to the pre-assessment team
		Postal contact	Collection of postoperative complication data through postal survey

Adapted from Shojania et al. [24] Closing the Quality Gap: A Critical Analysis of Quality Improvement Strategies (Vol. 2: Diabetes Mellitus Care). Technical Reviews, Rockville (MD): Agency for Healthcare Research and Quality (US). *LOS* length of stay, *MEWS* Modified Early Warning System, *BP* blood pressure, *WHO* World Health Organization, *SMS* Short Message Service.

• All epubs ahead of print which are indexed in one of the selected databases by the end date specified for the review

• QI papers reporting upon a deliberate effort to produce change in perioperative care. This may be in the form of a QI report, or a study of a QI method or quality intervention

This review will exclude

• Audits, unless they explicitly report on the implementation of a QI method which is designed to produce and evaluate a change

• Qualitative papers reporting exclusively on staff or patient experience of using QI methods

• Papers reporting on screening programmes and diagnostic interventions such as endoscopy and end-of-life care

• Papers reporting on secondary analyses where the main results have been published elsewhere

• Editorials and articles not published in the English language

• Abstracts and conference proceedings

Disagreements about eligibility will be resolved by discussion within the team.

### Search strategy

Databases will be selected for their ability to represent surgical and improvement method literature. Searches will be performed in the Ovid SP version of Medline, Scopus, the Cochrane Central Register of Controlled Trials, the Cochrane Effective Practice and Organisation of Care (EPOC) database (which indexes interventional studies focused on improvement in healthcare delivery) and the related articles function of PubMed. The journal *BMJ Quality* will be searched online using the find function for perioperative and surgical terms. MeSH terms, search terms, thesaurus mapping and Boolean operators will be used.

**Figure 1:**
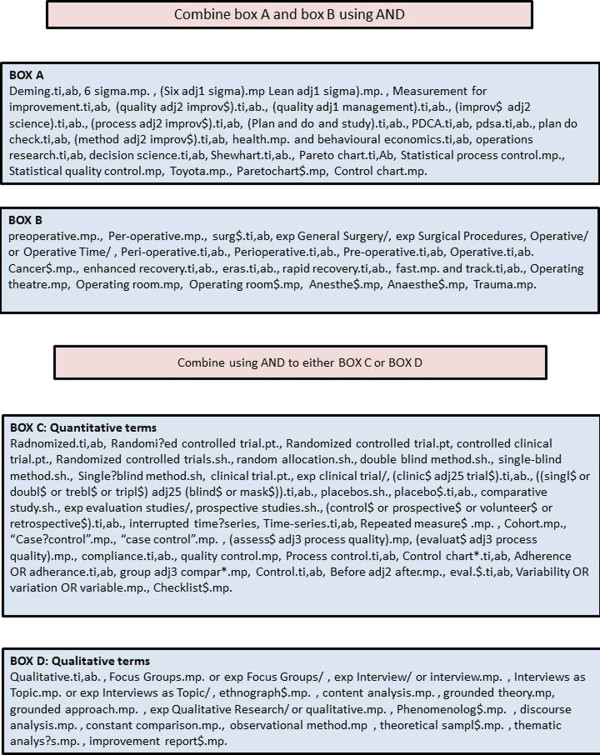
Search strategy.

The search strategy (Figure 1) was designed by three reviewers (ELJ, MDW and GPM). It will be conducted by an experienced research fellow (ELJ) who will apply the restrictions of publication year (2000–2014), humans NOT animals, and NOT infants. The search strategy is intended to capture terms relating to (i) surgery, (ii) quality improvement and (iii) methodology. Improvement terms were adapted from the improvement science research scan produced by the Health Foundation [1].

A training exercise was undertaken with a sample of search results where two authors (ELJ and MDW) considered selected full-text articles and discrepancies were resolved with a third reviewer (GPM). This enabled the authors to refine inclusion and exclusion criteria, ensuring consensus and reliable article selection.

A further training exercise will be undertaken whereby two authors (ELJ and NJL) will independently rate a sample of full-text articles against the TIDieR checklist until a high agreement is reached. TIDieR [4] is recommended by the Enhancing the Quality and Transparency of Health Research (EQUATOR) Network as an extension of the Consolidated Standards of Reporting Trials (CONSORT) [25] and SPIRIT [26] statements to improve reporting across all ‘evaluative’ study designs. Each item in the checklist will have an explanatory statement to guide the rater on how it should be interpreted. In accordance with previous work conducted by Hoffman and colleagues [12], the 12 items on the TIDieR checklist will be rated as ‘Yes’ (indicating that the description of that element of the intervention had been explicit) or ‘No’ (not reported or not clearly described).

Two reviewers (ELJ and NJL) will independently assess titles and abstracts of all abstracts to select and obtain full-text articles. The search results will be supplemented with hand searching of the reference lists of the full-text articles and of one recently published systematic review on improvement science [2] (ELJ).

### Data extraction

This review defines a quality intervention as a change to process directed at securing improvement, for example, introducing joint working patterns at weekends for assistant practitioners and foundation year 1 doctors to improve the rate of peripheral cannula insertion to reduce missed antibiotics. A QI method is defined as the process by which the change is supported and facilitated, for example, PDSA cycles. Patient and public involvement will be defined as the incorporation of the knowledge, skills and experience of patients, carers and the public into a study [27,28].

Data will be extracted from each selected paper by ELJ and NJL using standardised Excel templates. The first template will contain the 12 TIDieR checklist items [4] for recording the description of the intervention. The second template will contain key elements of the QI method. For purposes of this analysis, the salient features of quality improvement methods were determined by the authors following review and discussion of the relevant literature (see Table 2 for the list of features). When reports contain descriptions of two interventions, they will be rated separately. Checklists have also been published to facilitate reporting of PPI [21,29]. However, the emphasis of this review is upon the completeness of the QI reporting so the papers will be judged against only one PPI criterion, scoring ‘DONE’ if patient involvement is specified, ‘NOT CLEAR’ if it is not reported and ‘NOT DONE’ if patients are explicitly not involved. Discrepancies in the allocation of checklist scores will be resolved by discussion with a third reviewer (GPM or MDW).

**Table 2:** Data extraction template items

**Demographics**	**Quality intervention (TIDieR parameters)**	**QI method**
Author, year, country, surgical speciality	1. Brief name	1. Sample size
	2. Why (rationale for intervention)	2. Baseline measurement
	3. What (materials used to apply the intervention)	3. Data collection schedule
	4. Procedures (processes used in the intervention)	4. Data analysis (e.g. driver diagrams)
	5. Who (who delivered the intervention, including level of training)	5. Data volume/duration (e.g. length of PDSA cycle)
	6. How (mode of delivery: face to face, internet)	6. Explicit description of prediction of change
	7. Where (location: emergency or elective, and primary or secondary care)	7. Missing data (and reasons given)
	8. When and how much (duration, dose, intensity)	8. Description of generalizability
	9. Tailoring (was the intervention planned to be personalised)	9. Adverse effects (on health care providers and resource utilisation)
	10. Modifications (describe what, why, when and how modifications were made)	10. Presence and type of patient or stakeholder involvement (collaborative or consultative)
	11. How well (strategies to improve or maintain compliance)	
	12. How well (outcome of compliance assessment)	

### Data analysis

Data will be analysed descriptively using an Excel data extraction sheet. Nominal data will be used to present the proportion of complete and incomplete TIDieR checklist items. The potential transferability of findings between contexts will be considered. This is a descriptive review, and meta-analysis will not be undertaken.

Consistent with the principle that reviews may engage an iterative process [30], the review may evolve iteratively to include additional analysis such as bibliometric measures and descriptions of the fidelity of the interventions.

## Discussion

This review has a number of strengths and limitations. It will be the first review assessing how well QI methods and quality interventions are described across diverse settings (emergency and elective, and primary and secondary care) in perioperative care. This will advance understanding on what is required to improve reporting on QI methods and quality interventions. The review will extend beyond a presentation of raw outcome data, also considering the following: What rationales are provided for the application of specific QI strategies? How is QI defined? To what extent are patients involved? The findings of this review will be used to generate a research protocol to identify and resolve the challenges associated with defining and providing accounts of all of the elements of QI methods and interventions in surgery. This knowledge will generate a practical framework to facilitate the replication of effective QI strategies in practice. This framework will be pilot tested to confirm that it is a reliable method of specifying and describing the elements of QI methods and quality interventions. We anticipate that this work will be relevant to a wide multi-disciplinary community of clinicians and researchers who wish to reliably accelerate positive changes to practice to improve quality of care for patients and to improve the quality of the reporting of QI methods and interventions in perioperative literature.

Our study may have limitations. Papers not published in English will be excluded due to resource limitations, which may introduce bias. However, research has suggested that such exclusions tend to have a limited effect overall on systematic review conclusions [31]. Steps have been taken to limit potential subjectivity in data analysis by including standardised data extraction tools and checklists and by achieving consensus with a third reviewer. A team of social scientists and clinicians will ensure that key messages most appropriate to a surgical audience will be disseminated, addressing gaps in the current reporting of QI methods and interventions.

## Abbreviations

ELJ: Emma Jones; EPOC: Cochrane Effective Practice and Organisation of Care; GPM: Graham Martin; GRIPP: Guidance for Reporting Involvement of Patients and Public checklist; MDW: Mary Dixon-Woods; NJL: Nicholas Lees; PDSA: Plan-Do-Study-Act; PPI: Patient and public involvement; TIDieR: Template for Intervention Description and Replication; QI: Quality improvement.

## Competing interests

The authors declare that they have no competing interests.

## Authors’ contributions

MDW, GPM and ELJ conceived the idea for this study. ELJ developed the methods, and together with MDW, GPM, and NJL drafted this protocol. NJL acted as the second reviewer. All authors read and approved the final manuscript and have given final approval of the article to be published.
